# The Relations between the Oral Cavity and the General System

**Published:** 1897-12

**Authors:** 


### The Relations Between the Oral Cavity and the General System.

Remarks at Southern Dental Association, Old Point Comfort, Va., August 1897.

Dr. Walker, a Virginia physician, introduced by Dr. E. P. Beadles, who addressed the Association on the relationship between dentistry and general medicine. He said that the statement that most of the diseases to which humanity is liable have their origin in the stomach, is true to a certain extent. Ptyalin, the active principle of the saliva being an essential factor in digestion, the thorough mastication of food is very important, as digestion cannot be properly carried on if defective teeth interfere with mastication, preventing the proper admixture of ptyalin.

Again, when the teeth and gums are diseased food is taken into the stomach loaded with bacteria which find in the alimentary canal abundance of pabulum for their continued existence and increase. Thus an active element of disease is found in anto-infection from diseased teeth. Not infrequently, he said, his first step was to call in the services of the learned dentist whose assistance he had found invaluable. Many diseases—neurosis, abscesses, etc.,—demand the skill of the dentist rather than the learning of the physician.

Dr. H. E. Beach said this called to his mind a statement made by a learned professor in Philadelphia in 1869, who, in lecturing on "Diseases of the Alimentary Canal," said that in his opinion four-fifths of the human family died from some disease of the alimentary canal. To this, Dr. Beach added the statement, that in his opinion, and he did not think it could be successfully controverted—three-fourths of all the diseases of the alimentary canal have their origin in some pathological condition of the mouth. Thus, if this fact was recognized by physicians, three-fourths of four-fifths of the present mortality of the human race could be avoided or greatly lessened, by keeping the mouth in healthy condition.

